# The knowledge regarding the impacts and management of black triangles among dental professionals and laypeople

**DOI:** 10.1038/s41598-024-61356-0

**Published:** 2024-05-12

**Authors:** Mahmoud K. AL-Omiri, Danial Waleed Ahmad Atieh, Motasum Abu-Awwad, Abdullah A. Al Nazeh, Salem Almoammar, Saeed Awod Bin Hassan, Abdallah Ahmed Aljbab, Mohammed A. Alfaifi, Naji M. Shat, Edward Lynch

**Affiliations:** 1 Department of Fixed and Removable Prosthodontics, School of Dentistry, The University of Jordan, Queen Rania Street, Amman, 11942 Jordan; 2 Department of Prosthodontics, The City of London Dental School, Canada Water, Lower Road, London, UK; 3 Private Practice, Amman, Jordan; 4 Department of Pediatric Dentistry and Orthodontics Sciences, College of Dentistry, King Khalid University, Abha, Asir Saudi Arabia; 5 Department of Restorative Dental Sciences, College of Dentistry, King Khalid University, Abha, Asir Saudi Arabia; 6 Alqurayyat Specialized Dental Center, Ministry of Health, Al Qurayyat, Saudi Arabia; 7 Department of Prosthetic Dental Sciences, College of Dentistry, King Khalid University, Abha, Asir Saudi Arabia; 8 Department of Prosthodontics, Faculty of Dental and Oral Surgery, University of Palestine, Gaza, Palestine; 9 De Montfort University, Leicester, UK

**Keywords:** Black triangles, Interdental papillae, Gingival embrasure, Appearance, Knowledge, Satisfaction, Smile, Dental diseases, Restorative dentistry

## Abstract

This study aimed to assess the knowledge regarding impacts, causes and management of black triangles (BT) among participants from different educational backgrounds including dental students, dentists and laypeople. This descriptive cross-sectional observational research included 435 participants who comprised 4 groups: pre-clinical (3rd year) dental students, clinical (4th and 5th year) dental students, dentists, and laypeople. A constructed self-reported questionnaire was utilized to assess participants’ demographic data and their knowledge of the impacts, causes and management of BT. The VAS scale was used to assess participants’ ratings for the impacts of BT on esthetics, with 0 meaning no impact and 10 meaning very severe negative impacts. The most reported treatments for BT were “cannot be treated” 99.3% and “non-surgical periodontal treatment” 67.1%. Meanwhile, the least reported was “modify the porcelain” 41.8%. The most reported cause of BT was “periodontal disease” 85.1%. However, the least reported were “parafunction” and “deep implants” 33.1% each. Dental professionals had better knowledge of the causes (t = 8.189, P < 0.001) and management (t = 8.289, P < 0.001) of BT than the non-dental participants. The dentists had the best knowledge, while the laypeople had the least knowledge of the causes (F = 62.056, P < 0.001) and treatment (F = 46.120, P < 0.001) of BT. The knowledge of the causes (t = 0.616, P = 0.538) and treatment (t = 1.113, P = 0.266) for BT was not significantly different between males and females. Age was not significantly related to the total knowledge about the causes (r = −0.034, P = 0.475) or treatment (r = −0.034, P = 0.482) for BT. Dental professionals had better knowledge of the impacts, causes and management of BT than the non-dental participants. The dentists were the best, while the laypeople were the worst in this regard. Age and gender had no relationships with the knowledge of causes or management of BT.

## Introduction

The loss of interdental gingival papillary tissue results in the formation of a triangular space between the dentition known as open gingival embrasures or black triangles^1–4^. This might result in esthetic troubles, speech problems, food impaction and/or improper plaque control^5–7^. Black triangles, especially between the central incisors, are considered among the worst esthetic factors that negatively impact smile esthetics^8–12^.

The loss of support for interdental papillae is multifactorial, and would result from the loss of tooth contact, loss of bone, or increased distance from tooth contact point to the bony crest that is caused by several reasons, including periodontal disease, periodontal surgery, traumatic insults, improper tooth surface contours, aging, tooth spacing and loss of teeth^5,13,14^. Also, orthodontic treatment^15^ and implant restorations are associated with higher chances of papillary loss^16–18^.

Currently, management of black triangles includes prosthetic^14,19,20^, orthodontic^21^, and surgical approaches^22,23^ as well as tissue regeneration^24^ and tissue volumising^25^. Considering the difficulty in regenerating the interdental papillary tissue^26^, it is important to prevent black triangles by having enough support for the interdental papilla and not exceeding certain dimensions between the contact of teeth and the alveolar bone crest^5^. This is challenging as regeneration of lost tissue is difficult and requires maintaining the interdental papillary tissue volume within certain and difficult to obtain circumstances^8,26^. Also, having long interdental contact was preferred by patients as opposed to the presence of black triangles^8^.

Perception of esthetics is a complicated dynamic phenomenon affected by multiple dimensions including geographic, demographic (gender, age and education), socio-cultural and psychological factors^27–34^. Furthermore, previous research demonstrated significant differences between patients’ and dentists’ opinions regarding face and smile esthetics^35,36^.

Hence, dental professionals were found to be more critical in their judgment of dental and smile esthetics than laypeople^12,37–44^, and this might owe to their dental education^31^. In addition, dental specialists perceive the black triangle as less attractive than non-specialists or laypeople^28,37,39^. Moreover, younger patients and females perceive black triangles as less attractive than males and older patients^45,46^. Nevertheless, laypeople and periodontists were found to consider the inflamed gingiva as worse than black triangles^12^.

This potentially inspired investigators to better understand how to prevent and manage black triangles. In fact, a successful treatment would probably result when the goals and expectations of the patient and the clinician overlap^12,47^. This may help in directing the appropriate treatment to the patient and by this, save time, efforts, and costs^29,30,33,34^.

The literature lacks studies that investigate the knowledge of participants concerning the causes and management of black triangles. In addition, the literature lacks studies that explore the knowledge among different study groups including dentists, clinical dental students, preclinical dental students and laypeople. Furthermore, the literature is short in studies concerning the associations between knowledge regarding black triangles and age and gender.

Consequently, this study was conducted to explore the knowledge regarding the impacts, causes and management of black triangles among participants from different educational backgrounds. This could add further guidance to better understand the factors involved in the perception and management of black triangles.

The aim of the current study was to identify the knowledge regarding the impacts, causes and management of black triangles, and the relationship between the knowledge and the educational background among preclinical dental students, clinical dental students, dentists and laypeople.

The null hypothesis for this study was that there is no difference in the knowledge regarding the impacts, causes and management of black triangles between preclinical dental students, clinical dental students, dentists and laypeople.

## Materials and methods

### Study design and population

This descriptive cross-sectional, observational investigation was conducted between June 2022 and October 2022 in the University of Jordan considering the guidelines of the Helsinki Declaration (9th version, 2013). It was ethically approved by the Institutional Review Board (IRB) of the University of Jordan (Reference number: 19-2022-238 dated 17-4-2022). A signed written informed consent was provided by each participant before inclusion in the study.

The participants were invited to participate and were recruited from their laboratories (3rd year pre-clinical dental students), clinics (4th and 5th year dental students), offices (employees) and practices (dentists).

Simple randomization utilizing computer generated numbers was used to select the place (laboratories, clinics, offices and practices) of recruitment. A non-probability, convenient, and purposive sampling was used to recruit the participants in this study.

The invitation to participate in this study was extended to 450 participants, and 435 accepted to participate and were recruited (response rate = 96.7%). The study sample consisted of 4 groups including 3rd year preclinical dental students, 4th and 5th year clinical dental students, dentists and laypeople.

The participants were included if they were able to comprehend the questionnaire, did not have debilitating disease or mental disorders and were able to provide a signed informed consent. Also, dentists were included if they are currently practicing dentistry and registered with the Jordan Dental Association.

Dentists were excluded if they were not practicing or not registered with the Jordan Dental Association. Also, participants with history of mental disorders or debilitating disease were excluded.

### Study instruments and procedures

After recruitment, the participants were requested to complete a constructed self-reported questionnaire. The questionnaire was adopted from Atieh (2023)^48^. The questionnaire was developed, used, and validated in a previous investigation^48^. The development of the questionnaire involved reviewing the relevant literature and drafting the available causes, impacts and management of black triangles. Then, a panel of dental professionals with previous experience with black triangles (4 prosthodontists, 2 periodontists, 2 orthodontists, 1 oral surgeon, and 2 general dental practitioners) was consulted regarding the developed and drafted causes, impacts and management of black triangles. They were requested to comment on the clarity of the drafted questionnaire as well as to add any missing causes, impacts and managements of black triangles. Following the feedback of the consulted dental professionals, a final draft was prepared and sent back to the consulted professionals for final suggestions. Then, the used questionnaire was finalized. After development, the questionnaire was used and validated in a previous investigation that started with a pilot study, which validated and tested the questionnaire for clarity and effectiveness^48^. In addition, the test–retest reliability was conducted by Atieh (2023)^48^ as well as during this investigation to indicate the reliability of the used questionnaire.

To assess the reliability of participants’ responses to the questions, forty participants (ten from each group) were asked to answer the questions twice with a one week interval between the two occasions. In this regard, the Kappa value ranged between 0.8 and 0.9 for the tested questions, indicating an adequate reliability.

The utilized questionnaire in this study included 4 parts. The first part included items to record the demographic data of the participants including gender, age, level of education, educational background, marital status, place of residence, income and experience for dentists.

The second part of the questionnaire included items to assess participants’ knowledge and awareness of black triangles in the everyday life, items to record whether the participants had previous experiences with black triangles, and items with VAS scales to measure their ratings for the impacts of black triangles on the esthetics and appearance of individuals.

The VAS scale was used to assess participants’ ratings for the impacts of black triangles on esthetics and the appearance of individuals, 0 meant no impact and 10 meant very severe negative impacts. The visual analogue scale (VAS) was used in this study because it is considered a simple, valid and reliable method for assessment^48–51^. Also, adequate level of reliability was shown for the VAS when was used in previous literature regarding the black triangles^48,51^.

The third part of the questionnaire included items to assess participants’ knowledge of the possible causes of black triangles. This part of the questionnaire assessed the participants’ knowledge of 11 investigated causes of black triangle. The participants were asked whether each one of the investigated causes could be a possible cause for black triangles or not. A total score of knowledge about causes of black triangles was calculated by denoting one for each correct answer selected by the participant (possible minimum score is 0 and possible maximum score is 11). The participants were also asked to report any other possible cause for black triangles that was not mentioned in the questionnaire.

The fourth part of the questionnaire included items to assess participants’ knowledge of the available management of black triangles. The study investigated the participants’ knowledge of 8 investigated treatments of black triangle. The participants were asked whether each one of the investigated managements could be a possible management for black triangles or not. A total score of knowledge about treatment of black triangles was calculated by denoting one for each correct answer selected by the participant (possible minimum score is 0 and possible maximum score is 8). The participants were also asked to report any other possible management for black triangles that was not mentioned in the questionnaire.

### Study outcome measures

The main outcome measures for this study were participants’ knowledge regarding impacts, causes, and management of black triangles, and the level of participants’ education. The secondary outcome measures were the relationship between participants’ demographics (age, gender, and educational background) and their knowledge regarding black triangles.

### Statistical analysis

The statistical analysis for this investigation was carried out utilizing the Statistical Package for Social Sciences (IBM SPSS Statistics v23.0; IBM Corp., USA). The data was examined for normal distribution and the proper statistical analyses tests were then utilized. The continuous data was expressed as means, standard errors, standard deviations and confidence intervals, meanwhile the categorical data was described as frequencies, percentages, medians, minimum, maximum and interquartile ranges.

Correlations between different variables parametric variables were tested utilizing the Pearson’s r test and the Point biserial correlation (r). The independent student t-test was used for two-group comparisons, and the one-way analysis of variance (ANOVA) test and Post hoc analyses were used for comparison between more than two groups. Comparisons for non-parametric dependent variables (each tested causes and treatments of black triangles) between dental and non-dental participants were done using the Chi Square test. In addition, two-step hierarchical multiple linear regression analyses were carried out to examine the predictive power of the group and being from dental or non-dental backgrounds on the level of knowledge regarding black triangles, while controlling for the age and gender of participants. The significance level was set as two-tailed with P < 0.05 and 95% confidence intervals for all the analyses executed.

The G*power program (version 3.1.9.7) was used to perform a priori power analysis to determine the appropriate sample size for this investigation. The ANOVA test for multiple independent variables was utilized with a total of 4 groups, a power of 0.80, a significance level of 0.05 and an effect size of 0.25 based on Alomari et al. 2022^12^. This estimated a sample size of 180 participants. Allowing for a potential attrition rate of 20%, a sample size of 220 subjects was approximated. The invitation to participate was extended to 450 individuals, and 435 participants responded and participated in this investigation (response rate = 96.7%) and were the same cohort of patients in a previous investigation^51^.

## Results

Overall, 435 participants (136 males (31.3%) and 299 females (68.7%)) were recruited, and had their data collected and analyzed. The participants’ mean age was 28 years old (SD =  ± 10 years, age range = 18–78 years, 95% CI = 27–29 years).

Table 1 demonstrates the distribution of participants’ demographic data in this study. The study sample comprised 4 groups: dentists (n = 110), pre-clinical (3rd year) dental students (n = 104), clinical (4th and 5th year) dental students (n = 110) and laypeople (n = 111) (Table 1).

**Table 1:** The distribution of the categorical demographic data among the study population.

Categorical data and variables		Overall (n = 435)	Dentists (n = 110)	Clinical students (n = 110)	Pre-clinical students (n = 104)	Laypeople (n = 111)
Age	Mean ± SD	28.30 ± 9.99	34.10 ± 9.03	22.26 ± 1.14	21.03 ± 0.897	35.33 ± 11.85
Gender n (%)	Male	136 (31.3)	34 (30.9)	33 (30.0)	34 (32.7)	35 (31.5)
	Female	299 (68.7)	76 (69.1)	77 (70.0)	70 (67.3)	76 (68.5)
Marital status n (%)	Single	310 (71.3)	46 (41.8)	106 (96.4)	104 (100.0)	54 (48.6)
	Married	125 (28.7)	64 (58.2)	4 (3.6)	0 (0.0)	57 (51.4)
Educational background n (%)	Dental	324 (74.5)	110 (100.0)	110 (100.0)	104 (100.0)	0 (0.0)
	Non-dental	111 (25.5)	0 (0.0)	0 (0.0)	0 (0.0)	111 (100.0)
Education level (dental) n (%)	Dental student	214 (49.2)	0 (0.0)	110 (100.0)	104 (100.0)	0 (0.0)
	Bachelors	41 (9.4)	41 (37.3)	0 (0.0)	0 (0.0)	0 (0.0)
	Higher studies	69 (15.9)	69 (62.7)	0 (0.0)	0 (0.0)	0 (0.0)
Education level (non-dental) n (%)	Diploma	37 (8.5)	0 (0.0)	0 (0.0)	0 (0.0)	37 (33.3)
	Bachelors	59 (13.6)	0 (0.0)	0 (0.0)	0 (0.0)	59 (53.2)
	Higher studies	15 (3.4)	0 (0.0)	0 (0.0)	0 (0.0)	15 (13. 5)

### General awareness of BT and knowledge of BT impacts on smile attractiveness

Table 2 shows the participants’ general awareness of black triangles and their knowledge of the significance and impacts of black triangles on smile attractiveness among the study sample. The dentists reported the highest general awareness of black triangles whilst the laypeople reported the least general awareness of the problem. The VAS scores for rating the impacts of black triangles on the esthetics and appearance of individuals was significantly different between groups (F = 3.769, P = 0.011). Further comparisons using Tukey post hoc test revealed that dentists (mean difference = −0.8119, P = 0.014) and clinical dental students (mean difference = −0.7337, P = 0.030) reported more negative impacts of black triangles on esthetics and appearance than laypeople.

**Table 2:** Participants’ knowledge and awareness of black triangles and their impacts on smile attractiveness (total n = 435).

Question	Descriptive		Total sample	G1 (n = 110)	G2 (n = 110)	G3 (n = 104)	G4 (n = 111)	P value
Heard about BT	No (n (%))		49 (11.3)	1 (0.9)	1 (0.9)	4 (3.8)	43 (38.7)	< 0.001^$^
	Yes (n (%))		386 (88.7)	109 (99.1)	109 (99.1)	100 (96.2)	68 (61.3)	
Saw BT between natural teeth	Never (n (%))		31 (7.1)	2 (1.8)	7 (6.4)	6 (5.8)	16 (14.4)	< 0.001^$^
	Rarely (n (%))		90 (20.7)	13 (11.8)	21 (19.1)	34 (32.7)	22 (19.8)	
	Sometimes (n (%))		251 (57.7)	68 (61.8)	69 (62.7	55 (52.9)	59 (53.2)	
	Usually (n (%))		55 (12.6)	23 (20.9)	11 (10.0	9 (8.7)	12 (10.8)	
	Always (n (%))		8 (1.8)	4 (3.6)	2 (1.8)	0 (0.0)	2 (1.8)	
Saw BT between dental prosthesis	Never (n (%))		44 (10.1)	2 (1.8)	3 (2.7)	9 (8.7)	30 (27.0)	< 0.001^$^
	Rarely (n (%))		93 (21.4)	11 (10.0)	24 (21.8)	34 (32.7)	24 (21.6)	
	Sometimes (n (%))		171 (39.3)	45 (40.9)	45 (40.9)	46 (44.2)	35 (31.5)	
	Usually (n (%))		120 (27.6)	50 (45.5)	36 (32.7)	15 (14.4)	19 (17.1)	
	Always (n (%))		7 (1.6)	2 (1.8)	2 (1.8)	0 (0.0)	3 (2.7)	
Impacts of the loss of interdental gingiva on esthetics	Mean VAS (SD)		7.8 (2.0)	7.9 (1.9)	8.1 (1.5)	7.8 (2.2)	7.6 (2.2)	0.158^#^
	Variance		3.9	3.6	2.2	4.9	4.8	
	Min–Max		0.0–10.0	0.0–10.0	5.0–10.0	0.0–10.0	0.0–10.0	
	Percentiles	25	7.0	7.0	7.0	7.0	7.0	
		50	8.0	8.0	8.0	8.0	8.0	
		75	10.0	10.0	10.0	10.0	10.0	
	95% CI		7.7–8.0	7.6–8.3	7.9–8.4	7.3–8.2	7.1–8.0	
Impacts of the black triangles on esthetics	Mean VAS (SD)		7.8 (2.0)	8.1 (1.7)	8.1 (1.7)	7.9 (2.1)	7.3 (2.4)	0.011^#^
	Variance		4.1	3.0	3.0	4.2	5.7	
	Min–Max		0.0–10.0	1.0–10.0	0.0–10.0	1.0–10.0	0.0–10.0	
	Percentiles	25	7.0	7.0	7.0	7.0	6.0	
		50	8.0	8.0	8.0	8.0	8.0	
		75	10.0	10.0	9.0	10.0	9.0	
	95% CI		7.6–8.0	7.8–8.4	7.7–8.4	7.5–8.3	6.9–7.8	

G1 = Dentists, G2 = clinical (4th and 5th year) dental students, G3 = Pre-clinical (3rd year) dental students, G4 = Laypeople, VAS = Visual analogue scale, SD = standard deviation, CI = confidence interval, P^$^ = Significance of the difference between groups utilizing Chi square test, P^#^ = Significance of the difference between groups utilizing ANOVA test.

Dentists, clinical and preclinical dental students heard more about black triangles than laypeople (P < 0.05, Table 3). Dentists saw more BT between teeth and prosthesis than clinical dental students, preclinical students and laymen (P < 0.05, Table 3). Clinical dental students saw more BT between dental prosthesis than preclinical dental students and laymen (P < 0.001, Table 3).

**Table 3:** Comparison between groups regarding the encounter of participants with black triangles (n = 435).

Item	Compared groups	MWU	Z	P
Heard about BT	Dentist versus Clinical dental students	6050.0	0.000	1.000
	Dentist versus Pre-clinical dental students	5552.0	−1.418	0.156
	Dentist versus Laypeople	3795.5	−7.026	< 0.001**
	Clinical versus Pre clinical dental students	5552.0	−1.418	0.156
	Clinical dental students versus Laypeople	3795.5	−7.026	< 0.001**
	Pre-clinical dental students versus Laypeople	3758.0	−6.172	< 0.001**
Saw BT between natural teeth	Dentist versus Clinical dental students	4822.0	−3.002	0.003*
	Dentist versus Pre-clinical dental students	3840.5	−4.653	< 0.001**
	Dentist versus Laypeople	4444.0	−3.903	< 0.001**
	Clinical versus Pre-clinical dental students	5552.0	−1.418	0.156
	Clinical dental students versus Laypeople	3795.5	−7.026	< 0.001**
	Pre-clinical dental students versus Laypeople	5605.5	−0.401	0.689
Saw BT between dental prosthesis	Dentist versus Clinical dental students	4986.5	−2.418	0.016*
	Dentist versus Pre-clinical dental students	3142.0	−6.051	< 0.001**
	Dentist versus Laypeople	3356.5	−6.045	< 0.001**
	Clinical versus Pre-clinical dental students	4150.5	−3.676	< 0.001**
	Clinical dental students versus Laypeople	4174.5	−4.227	< 0.001**
	Pre-clinical dental students versus Laypeople	5214.5	−1.277	0.202

BT = Black triangles, MWU = Mann Whitney U test statistic, Z = Z statistic, P = Probability value, ** = Probability values are statistically significant at 0.01 level, * = Probability values are statistically significant at 0.05 level.

### Knowledge of the causes and treatment of BT amongst the participants

Table 4 demonstrates the distribution of the knowledge regarding the causes and treatment of black triangles amongst the study participants. The most reported cause of black triangles among the study sample was “periodontal disease” (n = 370) followed by “bone loss” (n = 232). However, the least reported cause of black triangles was the “increased overjet/overbite” (n = 76) followed by “parafunction” and “deep implants” (n = 144 each) (Table 4). Meanwhile, the most reported treatment for black triangles among the study sample was “cannot be treated” (n = 432) followed by “non-surgical periodontal treatment” (n = 292). However, the least reported treatment for black triangles was “surgery without bone graft” (n = 106) followed by “removing implants” (n = 112) (Table 4).

**Table 4:** Distribution of the knowledge regarding the causes of black triangles among the study sample (n = 435).

Knowledge about	Response	Total sample		G1 (n = 110)		G2 (n = 110)		G3 (n = 104)		G4 (n = 111)		P
		n	%	n	%	n	%	n	%	n	%	
*Causes of black triangles*												
Bone loss	False	203	46.7	20	18.2	28	25.5	72	69.2	83	74.8	< 0.001**
	True	232	53.3	90	81.8	82	74.5	32	30.8	28	25.2	
Periodontal disease	False	65	14.9	5	4.5	9	8.2	9	8.7	42	37.8	< 0.001**
	True	370	85.1	105	95.5	101	91.8	95	91.3	69	62.2	
Violation of biologic width	False	231	53.1	27	24.5	30	27.3	80	76.9	94	84.7	< 0.001**
	True	204	46.9	83	75.5	80	72.7	24	23.1	17	15.3	
Insufficient contact point to bone crest distance	False	233	53.6	32	29.1	49	44.5	63	60.6	89	80.2	< 0.001**
	True	202	46.4	78	70.9	61	55.5	41	39.4	22	19.8	
Parafunction	False	291	66.9	62	56.4	80	72.7	76	73.1	73	65.8	0.029*
	True	144	33.1	48	43.6	30	27.3	28	26.9	38	34.2	
Ageing	False	239	54.9	49	44.5	66	60.0	47	45.2	77	69.4	< 0.001**
	True	196	45.1	61	55.5	44	40.0	57	54.8	34	30.6	
Increased overjet/overbite	False	368	84.6	84	76.4	90	81.8	97	93.3	97	87.4	0.005*
	True	67	15.4	26	23.6	20	18.2	7	6.7	14	12.6	
Over-contoured restorations	False	281	64.6	52	47.3	59	53.6	80	76.9	90	81.1	< 0.001**
	True	154	35.4	58	52.7	51	46.4	24	23.1	21	18.9	
Under-contoured restorations	False	290	66.7	68	61.8	62	56.4	71	68.3	89	80.2	0.001*
	True	145	33.3	42	38.2	48	43.6	33	31.7	22	19.8	
Deep implants	False	291	66.9	57	51.8	66	60.0	78	75.0	90	81.1	< 0.001**
	True	144	33.1	53	48.2	44	40.0	26	25.0	21	18.9	
Insufficient Implant-Implant or Implant-Tooth distance	False	272	62.5	40	36.4	55	50.0	88	84.6	89	80.2	< 0.001**
	True	163	37.5	70	63.6	55	50.0	16	15.4	22	19.8	
*Treatment of black triangles*												
Change restoration	False	180	41.4	21	19.1	27	24.5	55	52.9	77	69.4	< 0.001**
	True	255	58.6	89	80.9	83	75.5	49	47.1	34	30.6	
Modify the restoration	False	221	50.8	45	40.9	53	48.2	49	47.1	74	66.7	0.001*
	True	214	49.2	65	59.1	57	51.8	55	52.9	37	33.3	
Modify the porcelain	False	253	58.2	50	45.5	48	43.6	69	66.3	86	77.5	< 0.001**
	True	182	41.8	60	54.5	62	56.4	35	33.7	25	22.5	
Non-surgical periodontal treatment	False	143	32.9	27	24.5	29	26.4	40	38.5	47	42.3	0.009*
	True	292	67.1	83	75.5	81	73.6	64	61.5	64	57.7	
Surgery with bone graft	False	231	53.1	35	31.8	45	40.9	70	67.3	81	73.0	< 0.001**
	True	204	46.9	75	68.2	65	59.1	34	32.7	30	27.0	
Surgery without bone graft	False	329	75.6	62	56.4	79	71.8	88	84.6	100	90.1	< 0.001**
	True	106	24.4	48	43.6	31	28.2	16	15.4	11	9.9	
Remove implants	False	323	74.3	70	63.6	75	68.2	88	84.6	90	81.1	0.001*
	True	112	25.7	40	36.4	35	31.8	16	15.4	21	18.9	
Cannot be treated	False	3	0.7	2	1.8	0	0.0	1	1.0	0	0.0	0.298
	True	432	99.3	108	98.2	110	100.0	103	99.0	111	100.0	

G1 = Dentists, G2 = clinical (4th and 5th year) dental students, G3 = Pre-clinical (3rd year) dental students, G4 = Laypeople, P = Probability value utilizing Kruskal Wallis test to compare groups, ** = Probability values are statistically significant at 0.01 level, * = Probability values are statistically significant at 0.05 level.

Table 5 shows the presence of significant differences in participants’ knowledge about the causes (F = 62.056, P < 0.001) and treatment (F = 46.120, P < 0.001) of black triangles between the study groups. Further comparisons using the Scheffe Post hoc test revealed that dentists have better knowledge about the causes of black triangles than clinical dental students, pre-clinical dental students and laypeople (P < 0.05, Table 5). Similarly, clinical dental students had better knowledge about the causes of black triangles than pre-clinical dental students and laypeople (P < 0.001, Table 5). As well, pre-clinical dental students had better knowledge about the causes of black triangles than laypeople (P = 0.037, Table 5). Furthermore, dentists had better knowledge regarding the treatment for black triangles than pre-clinical dental students and laypeople (P < 0.001, Table 5). Similarly, clinical dental students had better knowledge about the treatment for black triangles than pre-clinical dental students and laypeople (P < 0.001, Table 5).

**Table 5:** Distribution of the mean scores of participants’ knowledge of causes and treatment for black triangles and comparison of between the study groups (n = 435).

Knowledge	Study groups	Descriptive statistics		ANOVA test	
		M	SD	F	P
About causes	Total sample^#^	4.65	2.71	62.056	< 0.001**
	Dentists	6.49	2.55		
	Clinical dental students	5.60	2.44		
	Pre-clinical dental students	3.68	1.86		
	Laypeople	2.77	2.13		
About treatment	Total sample^#^	4.13	1.78	46.120	< 0.001**
	Dentists	5.16	1.88		
	Clinical dental students	4.76	1.58		
	Pre-clinical dental students	3.57	1.32		
	Laypeople	3	1.35		

	Compared groups			Scheffe Post hoc test	
				Mean diff	P
About causes	Dentists versus Clinical dental students			0.890	0.038*
	Dentists versus Pre-clinical dental students			2.808	< 0.001**
	Dentists versus Laypeople			3.716	< 0.001**
	Clinical versus Pre-clinical dental students			1.917	< 0.001**
	Clinical dental students versus Laypeople			2.825	< 0.001**
	Pre-clinical dental students versus Laypeople			0.907	0.037*
About treatment	Dentists versus Pre-clinical dental students			1.586	< 0.001**
	Dentists versus Laypeople			2.163	< 0.001**
	Clinical versus Pre-clinical dental students			1.186	< 0.001**
	Clinical dental students versus Laypeople			1.763	< 0.001**

M = Mean, SD = Standard deviation, ANOVA = One-way Analysis of variance test, F = F-statistic, Mean diff. = Mean difference, P = Probability value, ** = Probability values are statistically significant at 0.01 level, * = Probability values are significant at 0.05 level. ^#^The participants had low to moderate level of knowledge about the causes and moderate knowledge about the treatment of black triangles using the interquartile equation.

Furthermore, the participants with dental backgrounds (Mean = 5.31 ± 2.55) had better knowledge about the causes of black triangles (t = 8.189, P < 0.001) than the non-dental participants (Mean = 2.85 ± 2.25). Also, the participants with dental backgrounds (Mean = 4.53 ± 1.74) had better knowledge about the treatment for black triangles (t = 8.289, P < 0.001) than the non-dental participants (Mean = 3.05 ± 1.41).

Additionally, the dental participants demonstrated significantly better knowledge (P < 0.05, Table 6) regarding each tested cause of black triangles than the non-dental participants, except for “parafunction” (**χ**^**2**^ = 0.000, P = 0.989) and “increased overjet/overbite” (**χ**^**2**^ = 0.900, P = 0.343). Furthermore, the dental participants demonstrated significantly better knowledge (P < 0.05, Table 6) regarding each tested type of treatment for black triangles than the non-dental participants, except for “removing implants” (**χ**^**2**^ = 3.314, P = 0.069) and “cannot be treated” (**χ**^**2**^ = 1.124, P = 0.289).

**Table 6:** Differences in the knowledge regarding each cause of black triangles based on being dental or non-dental participant (n = 435).

Knowledge about	Response	Dental		Non-dental		Chi-square
		n	%	n	%	χ^2^ (P)
*Causes of black triangles*						
Bone loss	False	115	36.3	88	74.6	50.677 (< 0.001**)
	True	202	63.7	30	25.4	
Periodontal disease	False	23	7.3	42	35.6	54.331 (< 0.001**)
	True	294	92.7	76	64.4	
Violation of biologic width	False	133	42.0	98	83.1	58.313 (< 0.001**)
	True	184	58.0	20	16.9	
Insufficient contact point to bone crest distance	False	139	43.8	94	79.7	44.340 (< 0.001**)
	True	178	56.2	24	20.3	
Parafunction	False	212	66.9	79	66.9	.000 (0.989)
	True	105	33.1	39	33.1	
Ageing	False	158	49.8	81	68.6	12.279 (< 0.001**)
	True	159	50.2	37	31.4	
Increased overjet/overbite	False	265	83.6	103	87.3	.900 (0.343)
	True	52	16.4	15	12.7	
Over-contoured restorations	False	186	58.7	95	80.5	17.924 (< 0.001**)
	True	131	41.3	23	19.5	
Under-contoured restorations	False	198	62.5	92	78.0	9.303 (0.002*)
	True	119	37.5	26	22.0	
Deep implants	False	195	61.5	96	81.4	15.287 (< 0.001**)
	True	122	38.5	22	18.6	
Insufficient Implant-Implant or Implant-Tooth distance	False	179	56.5	93	78.8	18.327 (< 0.001**)
	True	138	43.5	25	21.2	
*Treatment for black triangles*						
Change restoration	False	100	31.5	80	67.8	46.586 (< 0.001**)
	True	217	68.5	38	32.2	
Modify the restoration	False	143	45.1	78	66.1	15.160 (< 0.001**)
	True	174	54.9	40	33.9	
Modify the porcelain	False	164	51.7	89	75.4	19.830 (< 0.001**)
	True	153	48.3	29	24.6	
Non-surgical periodontal treatment	False	93	29.3	50	42.4	6.622 (0.010*)
	True	224	70.7	68	57.6	
Surgery with bone graft	False	144	45.4	87	73.7	27.660 (< 0.001**)
	True	173	54.6	31	26.3	
Surgery without bone graft	False	224	70.7	105	89.0	15.661 (< 0.001**)
	True	93	29.3	13	11.0	
Removing implants	False	228	71.9	95	80.5	3.314 (0.069)
	True	89	28.1	23	19.5	
Cannot be treated	False	3	0.9	0	0.0	1.124 (0.289)
	True	314	99.1	118	100.0	

χ^2^ = Chi-square statistic, P = Probability value using Chi-square test, ** = Probability values are statistically significant at 0.01 level, * = Probability values are statistically significant at 0.05 level.

However, no significant relationship was identified between participants’ age and the total knowledge about the causes (r = −0.034, P = 0.475) or the treatment (r = −0.034, P = 0.482) for black triangles. Besides, no significant differences were found between males and females regarding the knowledge of the causes (t = 0.616, P = 0.538) and treatment (t = 1.113, P = 0.266) for black triangles.

The two-step multiple hierarchical regression analyses showed that the group significantly contributed to the total knowledge regarding the causes of black triangles (R^2^ = 0.296, R^2^ change = 0.290, B = −1.361, β = −0.566, t = −8.432, P < 0.001, 95% CI of B = −1.678 to −1.061). Being a dentist was associated with 1.361 higher odds of having better knowledge regarding the treatment of black triangles than clinical dental students, 2.72 higher odds than preclinical dental students, and 4.08 higher odds than laypeople.

Also, the group significantly contributed to the total knowledge regarding the treatment of black triangles (R^2^ = 0.238, R^2^ change = 0.234, B = −0.770, β = −0.486, t = −6.961, P < 0.001, 95% CI of B = −0.987 to −0.552). Being a dentist was associated with 0.77 higher odds of having better knowledge regarding the treatment of black triangles than clinical dental students, 1.54 higher odds than preclinical dental students, and 2.31 higher odds than laypeople.

## Discussion

The results of this study revealed the existence of associations between the knowledge regarding black triangles and the study group as well as being from dental or non-dental background. Consequently, the null hypothesis was rejected.

The findings showed that the dentists had experienced more cases of black triangles in comparison to dental students and laypeople, possibly due to having higher experience and more practice experience. Also, participants with dental educational backgrounds heard more about black triangles than laypeople. This could be explained by the lack of exposure of laypeople to dental education compared to the other groups. Also, dentists and clinical dental students reported more negative impacts of black triangles on esthetics than laypeople.

This concurs with other findings showing that individuals with a dental background were more strict in their evaluation of different esthetic parameters than the laypeople^12,37–44,52–55^. However, this opposes other studies that could not find any difference^28,56–61^.

In contrast to this study, Kay et al. (2014) reported no difference in disutility perception between dental professionals and patients in relation to tooth loss^61^. They found that both dental professionals and patients value tooth loss similarly and reported more disutility as the missing teeth were nearer to the front of the mouth, except for the loss of the upper canine that was rated to cause more disutility by the dental professionals. This relates to the black triangles problem as both the loss of anterior teeth and black triangles cause spaces that lead to negative impacts on esthetics.

In addition, missing teeth would cause larger spaces than the ones that result from black triangles, and this might account for negatively perceiving the esthetics regardless being a dental professional or a patient. The differences in psychological, cultural, and social factors could also account for this contrast, as well as differences in the tested parameters and methodologies adopted during these studies.

Dental participants demonstrated better knowledge than the non-dental ones in most of the knowledge items related to the causes of and treatments for black triangles, which might be reflected by the dental education that they were exposed to.

The findings also demonstrated that the odds of having better knowledge regarding causes and treatment of black triangles were the best for dentists, followed by clinical dental students, then the preclinical dental students, and finally the laypeople. This may be explained as dentists had already completed their dental degree, and that clinical students were further ahead in their degree than the pre-clinical dental students, and so they were more likely to have gained greater knowledge related to the black triangles and be more educated than the other groups. In addition, the laypeople had no dental education in this regard which resulted in them having the least knowledge regarding the causes and treatment of black triangles. No studies could be found that compared those specific aspects, but in a similar manner, the study by Costa and colleagues found that dentists had greater knowledge about sedation, followed by dental students and laypeople^62^. Moreover, the work by Al-Omiri and his group found that students in the higher years had better knowledge about oral health^30,63^.

No differences were found between the male and female participants in this study, and no studies looking particularly at the knowledge about black triangles could be found, so studies about knowledge of other aspects of black triangles and esthetics would be referred to. For instance, studies have found opposing results, where females had better knowledge than males about the relation of sugar intake and dental caries^64–66^. Furthermore, females also had better knowledge about oral hygiene practices and oral health than males^30,66–68^. Those differences may be explained by that, in this study, different aspects were tested and, in addition, female participants were more represented in the sample, and this calls for cautious interpretation. Utilizing various methods to measure the knowledge and perception might also underline this contrast.

Also, some researchers investigated the perception of black triangles as well as other esthetic parameters and concluded that women were more judgmental in their evaluation of black triangles and perceived them as less attractive than men^46,69^. However, this does not agree with the results of other studies investigating different esthetic factors^12,50,70,71^. This might owe to variations in the methods used to evaluate perception, differences in tested esthetic parameters as well as the sample demographics and the number of female participants.

Furthermore, no significant relationships were identified between participants’ age and the total knowledge about the causes or the treatment for black triangles. This might be related to the exposure of individuals to social media and having information regardless of the age.

No studies were available to compare with in this regard, so comparison to studies that tested other aspects would be refereed to. For example, this does not agree with previous findings that younger dentists were more familiar with preventive measures than the older counterparts, which is because they were exposed to the more recent dental education curriculum that puts more emphasis on the preventive approaches^72^. Also, multiple studies have shown that older subjects are less critical when it comes to esthetics^30,46,73,74^. Nonetheless, this was not shown in other studies^12,71^. This contrast might be attributed to variations in evaluated age groups and sample demographics, differences in the evaluated aspects of esthetics, and differences in education.

The study limitations included that in the present study, racial, social and cultural factors were not considered during this study. Besides, the age and gender distribution were beyond control among some groups such as the dental students who had a small age range. However, careful interpretation of those factors was undertaken. In addition, the confounding effects of age and gender were considered in the hierarchical regression analysis. Furthermore, the responses to the study instrument were subjective and self-reported by the participants; however, the utilized questionnaire was simple, clear, easy to score, and the participants were well informed and had any query answered by the investigators. Also, the reliability of the items was tested and ensured. Furthermore, the participants were recruited from available locations, which may potentially limit the generalizability of the findings of the study.

More investigations are required to highlight the possible effects of cultural, social, personality and racial factors on the knowledge and perception of black triangles and the role of different educational backgrounds in this regard. Comparisons between participants from different social, cultural, and racial backgrounds would highlight the impacts of how black triangles are perceived by different populations, and provide an insight into a more holistic understanding of the black triangles problem. Evaluation of personality might also identify how various personality factors potentially impact the perception of black triangles. Also, further investigations using larger samples are advisable on different populations.

## Conclusions

Within the limitations of this research, it was concluded that dental professionals have more negative perception of the impacts of black triangles on esthetics than laypeople. In addition, having a dental educational background was associated with better knowledge about the impacts, causes and treatment of black triangles.

## Data Availability

Data generated and analysed during this study are available from the corresponding author upon request to the following email: alomirim@yahoo.co.uk.
